# Bis(benzyl­trimethyl­ammonium) dichromate(VI)

**DOI:** 10.1107/S1600536811043091

**Published:** 2011-10-22

**Authors:** Lei Jin, Ning Liu

**Affiliations:** a College of Chemistry and Chemical Engineering, Southeast University, Nanjing 210096, People’s Republic of China

## Abstract

The asymmetric part of the title compound, (C_10_H_16_N)_2_[Cr_2_O_7_], contains one cation and a half of the dichromate dianion, which has a staggered conformation and exhibits disorder of the bridging O atom around the inversion center over two positions in a 1:1 ratio. Weak inter­molecular C—H⋯O hydrogen bonds link cations and anions into a three-dimensional structure.

## Related literature

For related structure, see: Jin *et al.* (2011 ▶).
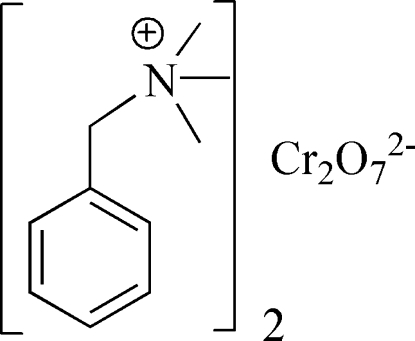


## Experimental

### 

#### Crystal data

(C_10_H_16_N)_2_[Cr_2_O_7_]
*M* *_r_* = 516.48
Monoclinic, 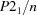

*a* = 8.8550 (18) Å
*b* = 12.442 (3) Å
*c* = 10.919 (2) Å
β = 91.75 (3)°
*V* = 1202.4 (4) Å^3^
*Z* = 2
Mo *K*α radiation
μ = 0.94 mm^−1^
*T* = 291 K
0.28 × 0.24 × 0.20 mm

#### Data collection

Rigaku Mercury2 (2x2 bin mode) diffractometer
Absorption correction: multi-scan (*CrystalClear*; Rigaku, 2005 ▶) *T*_min_ = 0.778, *T*_max_ = 0.834
10940 measured reflections
2356 independent reflections
2070 reflections with *I* > 2σ(*I*)
*R*_int_ = 0.035

#### Refinement

*R*[*F* ^2^ > 2σ(*F* ^2^)] = 0.040
*wR*(*F* ^2^) = 0.108
*S* = 1.07
2356 reflections
148 parameters
60 restraints
H-atom parameters constrained
Δρ_max_ = 0.42 e Å^−3^
Δρ_min_ = −0.50 e Å^−3^

### 

Data collection: *CrystalClear* (Rigaku, 2005 ▶); cell refinement: *CrystalClear*; data reduction: *CrystalClear*; program(s) used to solve structure: *SHELXS97* (Sheldrick, 2008 ▶); program(s) used to refine structure: *SHELXL97* (Sheldrick, 2008 ▶); molecular graphics: *SHELXTL* (Sheldrick, 2008 ▶); software used to prepare material for publication: *SHELXTL*.

## Supplementary Material

Crystal structure: contains datablock(s) I, global. DOI: 10.1107/S1600536811043091/cv5168sup1.cif

Structure factors: contains datablock(s) I. DOI: 10.1107/S1600536811043091/cv5168Isup2.hkl

Supplementary material file. DOI: 10.1107/S1600536811043091/cv5168Isup3.cml

Additional supplementary materials:  crystallographic information; 3D view; checkCIF report

## Figures and Tables

**Table 1:** Hydrogen-bond geometry (Å, °)

*D*—H⋯*A*	*D*—H	H⋯*A*	*D*⋯*A*	*D*—H⋯*A*
C1—H1*A*⋯O4^i^	0.96	2.58	3.308 (4)	133
C2—H2*A*⋯O1^ii^	0.96	2.59	3.467 (4)	153
C2—H2*B*⋯O2	0.96	2.53	3.416 (5)	154
C2—H2*C*⋯O4^iii^	0.96	2.42	3.294 (4)	152
C4—H4*B*⋯O4^iii^	0.97	2.52	3.393 (4)	149

Symmetry codes: (i) 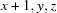; (ii) 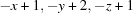; (iii) 
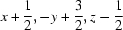
.

**Fig. 1.**
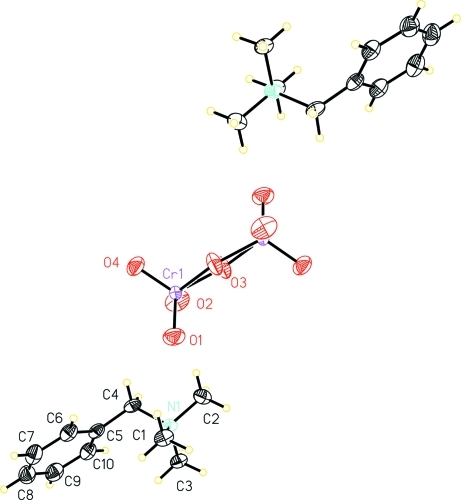
View of (I) showing the atomic numbering and 30% probability displacement ellipsoids. Unlabelled atoms are related to the labelled atoms by the (-x, 2 - y, 1 - z) symmetry transformation.

## supplementary crystallographic information

### Comment

In continuation of our structural study of organic-inorganic salts with benzyltrimethylammonium cations (Jin *et al.*, 2011), we present here the title compound, (I).

The  asymmetric unit of (I) consists of one half dichromate dianion and one benzyltrimethylammonium cation (Fig 1). The dichromate anion exhibits disorder of the bridging O3 atom around the invesion center over two positions in a ratio 1:1. The terminal bond distances of Cr–O are in the range 1.595 (3) - 1.610 (2) Å, and the bond angles of O–Cr–O vary in the range of 108.37 (17)–110.92 (14)°. The bridging Cr–O are in the range 1.754 (6) - 1.766 (6) Å, and the bond angles of O–Cr–O vary from 94.7 (2) to 120.51 (19) °. Atom O3 is disordered over two positions (separated by 0.794 (9) Å) in a ratio 1:1.

There are no classical hydrogen bonds in (I). The benzyltriethylammonium cations interact with the Cr_2_O_7_^2-^ anions through the weak intermolecular C–H···O hydrogen-bonded interactions (Table 1).

### Experimental

At room temperature, benzyltriethylammoniumchlorine (10 mmol, 2.28 g) was dissolved in 30 ml water, then a solution with (NH_4_)_2_Cr_2_O_7_ (5 mmol, 1.26 g) was dropped slowly into the solution with proper sirring. Yellow solid blocks appeared after several days (yield 75%). Single crystals suitable for X-ray structure analysis were obtained by the slow evaporation of the above solution after two weeks in air.

The dielectric constant of the compound as a function of temperature indicates that the permittivity is basically temperature-independent (ε = C/(T–T_0_)), suggesting that this compound is not ferroelectric or there may be no distinct phase transition occurring within the measured temperature (below the melting point).

### Refinement

H atoms were placed in calculated positions(C—H = 0.93-0.97 Å), and refined as riding, with *U*_iso_ = 1.2-1.5 *U*eq(C).

### Crystal data

**Table:**

(C_10_H_16_N)_2_[Cr_2_O_7_]	*Z* = 2
*M**_r_* = 516.48	*F*(000) = 540
Monoclinic, *P*2_1_/*n*	*D*_x_ = 1.427 Mg m^−^^3^
Hall symbol: -P 2yn	Mo *K*α radiation, λ = 0.71073 Å
*a* = 8.8550 (18) Å	θ = 3.0–26.0°
*b* = 12.442 (3) Å	µ = 0.94 mm^−^^1^
*c* = 10.919 (2) Å	*T* = 291 K
β = 91.75 (3)°	Block, orange
*V* = 1202.4 (4) Å^3^	0.28 × 0.24 × 0.20 mm

### Data collection

**Table:**

Rigaku Mercury2 (2x2 bin mode) diffractometer	2356 independent reflections
Radiation source: fine-focus sealed tube	2070 reflections with *I* > 2σ(*I*)
graphite	*R*_int_ = 0.035
Detector resolution: 13.6612 pixels mm^-1^	θ_max_ = 26.0°, θ_min_ = 3.0°
CCD_Profile_fitting scans	*h* = −10→10
Absorption correction: multi-scan (*CrystalClear*; Rigaku, 2005)	*k* = −15→15
*T*_min_ = 0.778, *T*_max_ = 0.834	*l* = −13→13
10940 measured reflections	

### Refinement

**Table:**

Refinement on *F*^2^	Primary atom site location: structure-invariant direct methods
Least-squares matrix: full	Secondary atom site location: difference Fourier map
*R*[*F*^2^ > 2σ(*F*^2^)] = 0.040	Hydrogen site location: inferred from neighbouring sites
*wR*(*F*^2^) = 0.108	H-atom parameters constrained
*S* = 1.07	*w* = 1/[σ^2^(*F*_o_^2^) + (0.057*P*)^2^ + 0.6088*P*] where *P* = (*F*_o_^2^ + 2*F*_c_^2^)/3
2356 reflections	(Δ/σ)_max_ = 0.001
148 parameters	Δρ_max_ = 0.42 e Å^−^^3^
60 restraints	Δρ_min_ = −0.50 e Å^−^^3^

### Special details

**Table:**

Geometry. All e.s.d.'s (except the e.s.d. in the dihedral angle between two l.s. planes) are estimated using the full covariance matrix. The cell e.s.d.'s are taken into account individually in the estimation of e.s.d.'s in distances, angles and torsion angles; correlations between e.s.d.'s in cell parameters are only used when they are defined by crystal symmetry. An approximate (isotropic) treatment of cell e.s.d.'s is used for estimating e.s.d.'s involving l.s. planes.
Refinement. Refinement of *F*^2^ against ALL reflections. The weighted *R*-factor *wR* and goodness of fit *S* are based on *F*^2^, conventional *R*-factors *R* are based on *F*, with *F* set to zero for negative *F*^2^. The threshold expression of *F*^2^ > σ(*F*^2^) is used only for calculating *R*-factors(gt) *etc*. and is not relevant to the choice of reflections for refinement. *R*-factors based on *F*^2^ are statistically about twice as large as those based on *F*, and *R*- factors based on ALL data will be even larger.

### Fractional atomic coordinates and isotropic or equivalent isotropic displacement parameters (Å^2^)

**Table:**

	*x*	*y*	*z*	*U*_iso_*/*U*_eq_	Occ. (<1)
C1	0.6409 (4)	0.8457 (3)	0.5418 (3)	0.0524 (7)	
H1A	0.7118	0.8025	0.5884	0.079*	
H1B	0.5473	0.8493	0.5838	0.079*	
H1C	0.6809	0.9168	0.5326	0.079*	
C2	0.5041 (4)	0.8658 (3)	0.3458 (3)	0.0558 (8)	
H2A	0.5448	0.9370	0.3392	0.084*	
H2B	0.4100	0.8687	0.3869	0.084*	
H2C	0.4877	0.8361	0.2653	0.084*	
C3	0.7592 (3)	0.7905 (2)	0.3526 (3)	0.0486 (7)	
H3A	0.7981	0.8618	0.3418	0.073*	
H3B	0.7423	0.7574	0.2739	0.073*	
H3C	0.8308	0.7487	0.4001	0.073*	
C4	0.5429 (3)	0.6849 (2)	0.4291 (3)	0.0470 (7)	
H4A	0.4491	0.6919	0.4721	0.056*	
H4B	0.5179	0.6583	0.3475	0.056*	
C5	0.6397 (3)	0.6029 (2)	0.4944 (3)	0.0456 (7)	
C6	0.6318 (4)	0.5878 (3)	0.6211 (3)	0.0541 (8)	
H6	0.5700	0.6321	0.6664	0.065*	
C7	0.7141 (4)	0.5085 (3)	0.6794 (3)	0.0649 (9)	
H7	0.7074	0.4997	0.7636	0.078*	
C8	0.8061 (4)	0.4422 (3)	0.6148 (4)	0.0681 (10)	
H8	0.8624	0.3890	0.6549	0.082*	
C9	0.8143 (4)	0.4550 (3)	0.4892 (4)	0.0670 (9)	
H9	0.8755	0.4096	0.4447	0.080*	
C10	0.7327 (4)	0.5344 (3)	0.4300 (3)	0.0537 (7)	
H10	0.7397	0.5424	0.3457	0.064*	
Cr1	0.10047 (4)	0.88613 (3)	0.53692 (4)	0.03659 (18)	
N1	0.6137 (2)	0.79616 (18)	0.4177 (2)	0.0407 (5)	
O1	0.2580 (3)	0.9159 (2)	0.6062 (2)	0.0755 (7)	
O2	0.1341 (4)	0.8252 (3)	0.4124 (2)	0.0863 (8)	
O3	0.0066 (8)	0.9953 (5)	0.4646 (4)	0.0720 (13)	0.50
O4	0.0014 (3)	0.8091 (2)	0.6192 (2)	0.0753 (7)	

### Atomic displacement parameters (Å^2^)

**Table:**

	*U*^11^	*U*^22^	*U*^33^	*U*^12^	*U*^13^	*U*^23^
C1	0.0550 (17)	0.0549 (18)	0.0474 (16)	−0.0100 (15)	0.0024 (13)	−0.0200 (14)
C2	0.0458 (17)	0.0575 (19)	0.064 (2)	0.0067 (14)	−0.0047 (14)	−0.0065 (15)
C3	0.0398 (14)	0.0510 (16)	0.0555 (17)	−0.0065 (13)	0.0098 (12)	−0.0085 (13)
C4	0.0387 (14)	0.0534 (17)	0.0487 (15)	−0.0164 (12)	−0.0005 (12)	−0.0095 (13)
C5	0.0472 (15)	0.0467 (16)	0.0428 (14)	−0.0196 (12)	0.0002 (12)	−0.0070 (12)
C6	0.0624 (19)	0.0548 (18)	0.0452 (16)	−0.0195 (15)	0.0041 (14)	−0.0092 (14)
C7	0.085 (2)	0.062 (2)	0.0471 (17)	−0.0263 (19)	−0.0090 (17)	0.0004 (15)
C8	0.076 (2)	0.053 (2)	0.075 (2)	−0.0156 (18)	−0.0153 (19)	0.0049 (17)
C9	0.071 (2)	0.0492 (19)	0.082 (2)	−0.0072 (16)	0.0116 (18)	−0.0101 (17)
C10	0.0634 (19)	0.0508 (17)	0.0474 (16)	−0.0124 (15)	0.0093 (14)	−0.0062 (14)
Cr1	0.0359 (3)	0.0317 (3)	0.0421 (3)	0.00047 (16)	−0.00114 (18)	0.00768 (16)
N1	0.0331 (11)	0.0450 (12)	0.0439 (12)	−0.0033 (9)	0.0015 (9)	−0.0107 (10)
O1	0.0554 (14)	0.0929 (18)	0.0772 (16)	−0.0188 (13)	−0.0147 (12)	0.0001 (14)
O2	0.0880 (18)	0.104 (2)	0.0677 (16)	−0.0161 (16)	0.0182 (14)	−0.0238 (15)
O3	0.090 (3)	0.056 (2)	0.070 (3)	0.030 (2)	0.003 (3)	0.015 (3)
O4	0.0664 (14)	0.0954 (18)	0.0642 (14)	−0.0221 (13)	0.0029 (12)	0.0330 (13)

### Geometric parameters (Å, °)

**Table:**

C1—N1	1.501 (3)	C5—C6	1.400 (4)
C1—H1A	0.9600	C6—C7	1.371 (5)
C1—H1B	0.9600	C6—H6	0.9300
C1—H1C	0.9600	C7—C8	1.370 (5)
C2—N1	1.504 (4)	C7—H7	0.9300
C2—H2A	0.9600	C8—C9	1.384 (5)
C2—H2B	0.9600	C8—H8	0.9300
C2—H2C	0.9600	C9—C10	1.373 (5)
C3—N1	1.492 (3)	C9—H9	0.9300
C3—H3A	0.9600	C10—H10	0.9300
C3—H3B	0.9600	Cr1—O2	1.593 (3)
C3—H3C	0.9600	Cr1—O4	1.595 (2)
C4—C5	1.498 (4)	Cr1—O1	1.610 (2)
C4—N1	1.527 (4)	Cr1—O3^i^	1.754 (6)
C4—H4A	0.9700	Cr1—O3	1.766 (6)
C4—H4B	0.9700	O3—O3^i^	0.794 (9)
C5—C10	1.392 (4)	O3—Cr1^i^	1.754 (6)
			
N1—C1—H1A	109.5	C8—C7—H7	119.7
N1—C1—H1B	109.5	C6—C7—H7	119.7
H1A—C1—H1B	109.5	C7—C8—C9	119.4 (4)
N1—C1—H1C	109.5	C7—C8—H8	120.3
H1A—C1—H1C	109.5	C9—C8—H8	120.3
H1B—C1—H1C	109.5	C10—C9—C8	120.4 (3)
N1—C2—H2A	109.5	C10—C9—H9	119.8
N1—C2—H2B	109.5	C8—C9—H9	119.8
H2A—C2—H2B	109.5	C9—C10—C5	120.9 (3)
N1—C2—H2C	109.5	C9—C10—H10	119.5
H2A—C2—H2C	109.5	C5—C10—H10	119.5
H2B—C2—H2C	109.5	O2—Cr1—O4	108.37 (16)
N1—C3—H3A	109.5	O2—Cr1—O1	109.21 (16)
N1—C3—H3B	109.5	O4—Cr1—O1	110.92 (14)
H3A—C3—H3B	109.5	O2—Cr1—O3^i^	120.51 (19)
N1—C3—H3C	109.5	O4—Cr1—O3^i^	101.8 (2)
H3A—C3—H3C	109.5	O1—Cr1—O3^i^	105.7 (3)
H3B—C3—H3C	109.5	O2—Cr1—O3	94.7 (2)
C5—C4—N1	115.2 (2)	O4—Cr1—O3	117.0 (3)
C5—C4—H4A	108.5	O1—Cr1—O3	115.0 (3)
N1—C4—H4A	108.5	O3^i^—Cr1—O3	26.1 (3)
C5—C4—H4B	108.5	C3—N1—C1	109.4 (2)
N1—C4—H4B	108.5	C3—N1—C2	109.3 (2)
H4A—C4—H4B	107.5	C1—N1—C2	108.6 (2)
C10—C5—C6	117.7 (3)	C3—N1—C4	111.0 (2)
C10—C5—C4	121.0 (3)	C1—N1—C4	110.7 (2)
C6—C5—C4	121.1 (3)	C2—N1—C4	107.8 (2)
C7—C6—C5	120.9 (3)	O3^i^—O3—Cr1^i^	77.8 (9)
C7—C6—H6	119.5	O3^i^—O3—Cr1	76.1 (8)
C5—C6—H6	119.5	Cr1^i^—O3—Cr1	153.9 (3)
C8—C7—C6	120.7 (3)		
			
N1—C4—C5—C10	−93.3 (3)	C5—C4—N1—C3	58.0 (3)
N1—C4—C5—C6	91.1 (3)	C5—C4—N1—C1	−63.7 (3)
C10—C5—C6—C7	0.4 (4)	C5—C4—N1—C2	177.7 (2)
C4—C5—C6—C7	176.2 (3)	O2—Cr1—O3—O3^i^	172.0 (11)
C5—C6—C7—C8	0.0 (5)	O4—Cr1—O3—O3^i^	58.6 (12)
C6—C7—C8—C9	−0.7 (5)	O1—Cr1—O3—O3^i^	−74.2 (11)
C7—C8—C9—C10	0.9 (5)	O2—Cr1—O3—Cr1^i^	172.0 (11)
C8—C9—C10—C5	−0.4 (5)	O4—Cr1—O3—Cr1^i^	58.6 (12)
C6—C5—C10—C9	−0.3 (4)	O1—Cr1—O3—Cr1^i^	−74.2 (11)
C4—C5—C10—C9	−176.0 (3)	O3^i^—Cr1—O3—Cr1^i^	0.0

Symmetry codes: (i) −*x*, −*y*+2, −*z*+1.

### Hydrogen-bond geometry (Å, °)

**Table:**

*D*—H···*A*	*D*—H	H···*A*	*D*···*A*	*D*—H···*A*
C1—H1A···O4^ii^	0.96	2.58	3.308 (4)	133.
C2—H2A···O1^iii^	0.96	2.59	3.467 (4)	153.
C2—H2B···O2	0.96	2.53	3.416 (5)	154.
C2—H2C···O4^iv^	0.96	2.42	3.294 (4)	152.
C4—H4B···O4^iv^	0.97	2.52	3.393 (4)	149.

Symmetry codes: (ii) *x*+1, *y*, *z*; (iii) −*x*+1, −*y*+2, −*z*+1; (iv) *x*+1/2, −*y*+3/2, *z*−1/2.
